# Development of low-cost micro-fabrication procedures for planar micro-thermoelectric generators based on thin-film technology for energy harvesting applications

**DOI:** 10.1371/journal.pone.0306540

**Published:** 2024-07-11

**Authors:** Sobhy M. Abdelkader, Donart Nayebare, Tamer F. Megahed, Ahmed M. R. Fath El-Bab, Mohamed A. Ismeil, Omar Abdel-Rahim

**Affiliations:** 1 Electrical Power Engineering, Egypt-Japan University of Science and Technology (E-JUST), Alexandria, Egypt; 2 Electrical Engineering Department, Faculty of Engineering, Mansoura University, Mansoura, Egypt; 3 Mechatronics and Robotics Engineering Department, Egypt-Japan University of Science and Technology (E-JUST), Alexandria, Egypt; 4 Electrical Engineering Department, Faculty of Engineering, King Khalid University, Abha, Saudi Arabia; 5 Electrical Engineering Department, Faculty of Engineering, Aswan University, Aswan, Egypt; Parul University Parul Institute of Technology, INDIA

## Abstract

With the rapid proliferation of portable and wearable electronics, energy autonomy through efficient energy harvesting has become paramount. Thermoelectric generators (TEGs) stand out as promising candidates due to their silent operation, high reliability, and maintenance-free nature. This paper presents the design, fabrication, and analysis of a micro-scale TEG for powering such devices. A planar configuration was employed for its inherent miniaturization advantages. Finite element analysis using ANSYS reveals that a double-layer device under a 50 K temperature gradient generates an impressive open-circuit voltage of 1417 mV and a power output of 2.4 μW, significantly exceeding its single-layer counterpart (226 mV, 0.12 μW). Validation against the analytical model results yields errors within 2.44% and 2.03% for voltage and power, respectively. Furthermore, a single-layer prototype fabricated using paper shadow masks and sputtering deposition exhibits a voltage of 131 mV for a 50 K temperature difference, thus confirming the feasibility of the proposed design. This work establishes a foundation for developing highly efficient micro-TEGs for powering next-generation portable and wearable electronics.

## 1. Introduction

Energy harvesting technology has recently emerged as a promising source of power for miniaturized and low-power electronic devices such as wireless sensor nodes [1] and wearable electronics [2]. Moreover, there is a growing demand for portable and wearable electronics over the past years [3]. This trend is accompanied by the next generation of artificial intelligence equipment and the Internet of Things (IoT) where people and things are connected anywhere at any time [4]. Portable and wearable electronic devices have a high potential for a wide range of applications, including health monitoring [5, 6], wireless communication [7], and environmental quality monitoring [8, 9]. Such applications require a stable, reliable, and durable power supply for proper functioning [10]. Batteries are presently the common power sources for these devices but must be replaced or recharged frequently, which is not only inconvenient and costly but also not always possible. For example, the case of implanted biomedical devices such as pacemakers that have 24/7 operation [2]. To overcome these constraints, researchers believe that developing a self-powered electrical energy harvesting system with high biocompatibility and durability would be a novel solution. Several energy scavenging techniques have been researched including piezoelectric [11, 12], triboelectric [13], and thermoelectric(TE) [14, 15]. Energy harvesting is an exciting field that has the potential to unlock a new source of renewable energy and contribute to a more sustainable future [16]. Moreso, thermoelectric energy harvesters have promising applications in the operation of miniature electronic devices since thermal energy is easily available. They have the ability to directly convert thermal energy into electricity and have the advantages of being silent, highly reliable, and requiring no maintenance. Their main disadvantage, however, is their low conversion efficiency. Their performance depends heavily on the dimensionless figure of merit (ZT) given by α^2^σT/k, where α is the Seebeck coefficient, σ is the electrical conductivity, T is the absolute temperature, while k is thermal conductivity. The goal is to increase α and σ while minimizing k to improve ZT and enhance the TEG performance. It is, however, difficult since the three parameters are interrelated and difficult to decouple [17]. As a result, several efforts have been made to improve these properties, ranging from organic materials like carbon nanotubes [18] to inorganic materials like nanowire silicon [19] and bismuth telluride [20] via nano engineering [21], band engineering [22], and defect engineering [23]. Less attention has been given to other strategies for improving the TEG performance, such as design optimization, increasing the integration density of thermoelectric pairs, and optimizing the temperature difference across the TEG. These play an equally important role in enhancing the output parameters of the module. Moreover, the output power is directly proportional to the number of pairs and the square of the temperature difference. In the current study, geometry and design optimization at the device level have been addressed. A double layer-double circuit planar design has been proposed to optimize the substrate and fabricate a high number of pairs for a given area. To boost the performance even further, the study includes strategies to improve heat transfer on the cold side to maximize the temperature difference. Thermoelectric devices are classified as planar or vertical based on the arrangement of the thermoelectric elements on the substrate and the direction of heat flow into the device [24, 25]. In vertical, the thermoelectric (TE) legs are orthogonal to the substrate, and the heat flow is vertical. This type of TEGs have low electrical resistance and no parasitic heat flow into the substrate. However, they are difficult to fabricate and less compatible with micro-electromechanical systems (MEMS) processes. On the other hand, Planar TEGs have TE legs that are laterally arranged on the substrate, and heat flow is lateral. They are easy to make because no bonding is required to connect the electrode to the TE legs, and they are suitable for low-cost mass production using thin-film technology. Planar TEGs also have the advantage of miniaturization which is significant for applications in portable and wearable electronic devices. Micro-fabrication using thin films involves the use of a pattern to develop and transfer a circuit onto a substrate through the lift-off process. The pattern is transferred onto the substrate using a shadow mask and a photoresist using photolithography. This process of patterning has been widely used in the area of micro structuring, but it is costly, very sensitive, and requires clean room conditions. The use of simple and easily available materials such as paper and acrylic to develop patterns for fabricating a micro-TEG is relatively new and has not been widely reported. Such options are simple and cheap to fabricate since they do not involve complex methods like photolithography and photoresist preparation. Additionally, they require no clean room conditions. The micro-patterns are designed and sketched in a simple 2D drawing software and then cut out on paper by direct laser writing using a Universal Laser System. With the masks ready, the process of fabricating the planar double-layer TEG was reduced to three major steps, while that of a single-layer planar type was only two major steps. This method of micro-fabrication is easy, cheap, repeatable, and could be adopted for mass production. In this work, the use of paper and acrylic to prepare shadow masks and fabricate a micro-TEG has been demonstrated with promising results. The main contribution of this study includes:

1. The paper proposes a new design for optimizing the performance of the micro-TEG with high integration of thermoelectric elements.
2. A simple and cheap micro-fabrication process involving the use of paper or acrylic for micro-patterning has been successfully demonstrated.

## 2. Concept of thermoelectric generation

### 2.1. Principle of operation and governing equations

According to the Seebeck effect, a TEG produces electricity directly from heat. Two different metals with opposite Seebeck effects are joined together at both ends. If subjected to a temperature difference, such that one side is heated and the other cooled, a voltage is induced. The magnitude of the induced voltage is proportional to the temperature difference.

**Fig 1:**
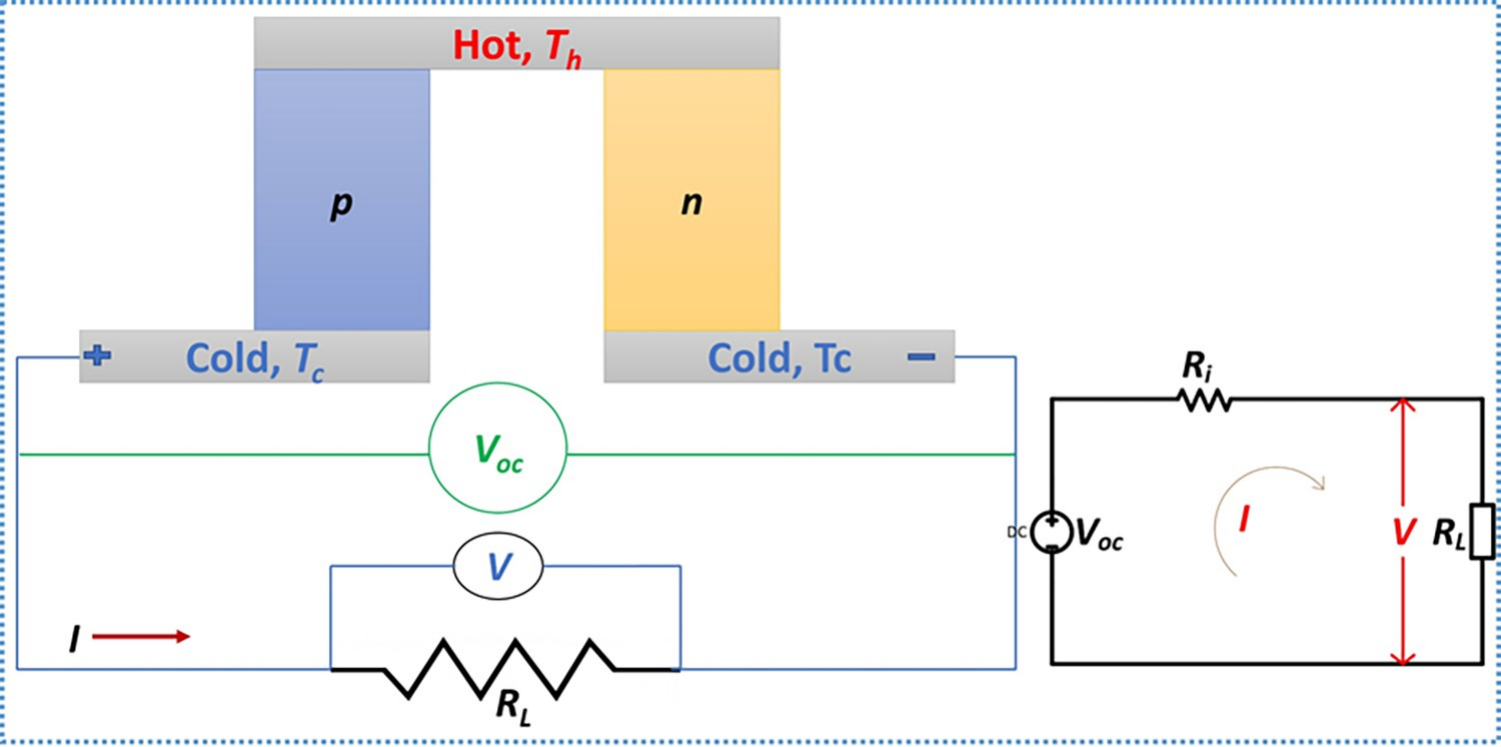
Schematic showing one thermoelectric pair (vertical configuration) and equivalent electrical circuit.

Consider one thermoelectric pair consisting of two different materials (i.e., p-type and n-type), as shown in Fig 1. When subjected to a temperature gradient, by heating one end and cooling the other, an emf is generated.

The thermoelectric open circuit voltage (V_oc_) induced is a function of the Seebeck coefficients of the thermoelectric materials and the temperature difference, given by the following expression [26]:

$$V_{ac}=(\alpha_{a}-\alpha_{b})(T_{h}-T_{c})=\alpha \Delta T$$ (1)

Where α_a_ and α_b_ are the Seebeck coefficients of p-type and n-type materials, α is the effective Seebeck coefficient of the pair while T_h_, T_c_, and *ΔT* are the hot side temperature, cold side temperature, and temperature difference respectively.

If an external load of resistance *R*_*L*_ is connected across the pair, as shown in Fig 1, the current flowing, *I* is given by:

$$I=\frac{V_{oc}}{R_{i}+R_{L}}$$ (2)

Where *R*_*i*_ is the steady-state internal electrical resistance of the pair, given by ρl/A. Where ρ is the electrical resistivity of the material, *l* is the length of the thermos element, and *A* is the cross-section area.

The load voltage (V) can be calculated from:

$$V=IR_{L}=V_{oc}-IR_{i}$$ (3)

Also given by:

$$V=\alpha \Delta T-IR_{i}$$ (4)

The power flowing through the load is then expressed as:

$$P_{L}={\left(\frac{V_{oc}}{R_{i}+R_{L}}\right)}^{2}R_{L}$$ (5)

For maximum power, the load resistance *R*_*L*_ must match the internal resistance of the generator (i.e., *R*_*L*_ = *R*_*i*_) [1, 27, 28]. Eq 5 then becomes:

$$P_{m}=\frac{{V_{oc}}^{2}}{4R_{i}}=\frac{{\left(\alpha \Delta T\right)}^{2}}{4R_{i}}$$ (6)

For a TEG of N pairs, the voltage and power of the module can then be computed as follows [26]:

$$V_{TEG}=N\alpha \Delta T$$ (7)

$$P_{TEG}=\frac{N{\left(\alpha \Delta T\right)}^{2}}{4R_{i}}$$ (8)

### 2.2. Device design

**Fig 2:**
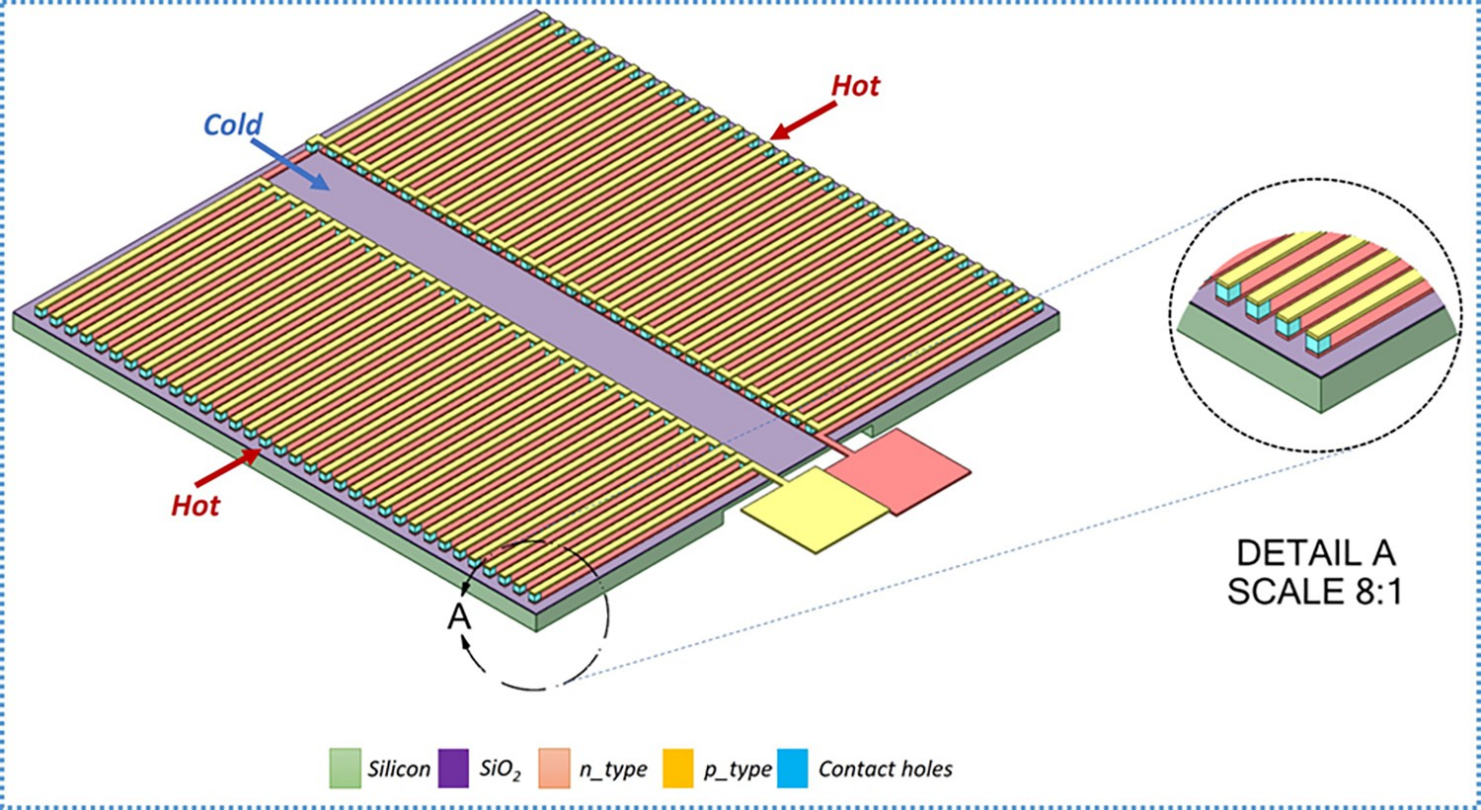
Isometric view of the proposed design.

The proposed design is a planar configuration with an array of thermocouples electrically connected in series and thermally in parallel. A double-layer architecture is utilized to optimize the substrate area and fabricate a high number of pairs per unit area. To further enhance the design, a double circuit is proposed as shown in Fig 2. The device is integrated on a silicon wafer substrate of thickness 500 μm. The p- and n-type elements are deposited in separate layers separated by a thin silicon dioxide insulating film and joined via contact holes. As shown in Fig 2, the hot junction is on either side of the substrate, while the cold junction is in the center. To improve heat exchange on the cold side, the substrate has a backside diaphragm located directly beneath the cold region. The device size is 35x35 mm2 with a thickness of 0.5 mm.

## 3. Finite element analysis

The proposed design was modeled and tested using the finite element method in the ANSYS 2020R2 model. Steady state thermal-electric analysis was performed to evaluate the open circuit voltage and temperature distribution of the proposed model. To define the boundary conditions and establish a thermal gradient between the hot and cold sides, the hot side temperature was first set at 308 K and the cold side at 298 K. The hot side temperature was varied to vary ΔT and simulate different practical applications. The model was categorized into 6 parts i.e., a silicon substrate, silicon dioxide insulating layer, n-type metal, p-type metal, contact holes, and the connecting pads. The mesh type and element size were manually inserted since the parts have different dimensions. The sweep method was selected for all the parts and an appropriate element size for each part was chosen. The mesh was of acceptable quality with 3829739 nodes and 512464 elements.

### 3.1. Temperature and voltage profiles

The open circuit voltage of one TE pair is directly proportional to the product of the effective Seebeck coefficient and the thermal gradient across the pair, as expressed in Eq 1. The voltage of one pair is very small and may not be usable for practical applications. To increase the output voltage, several pairs are connected in series. The total output voltage of the module is a function of the number of pairs, and this can be seen in Eq 7.

**Fig 3:**
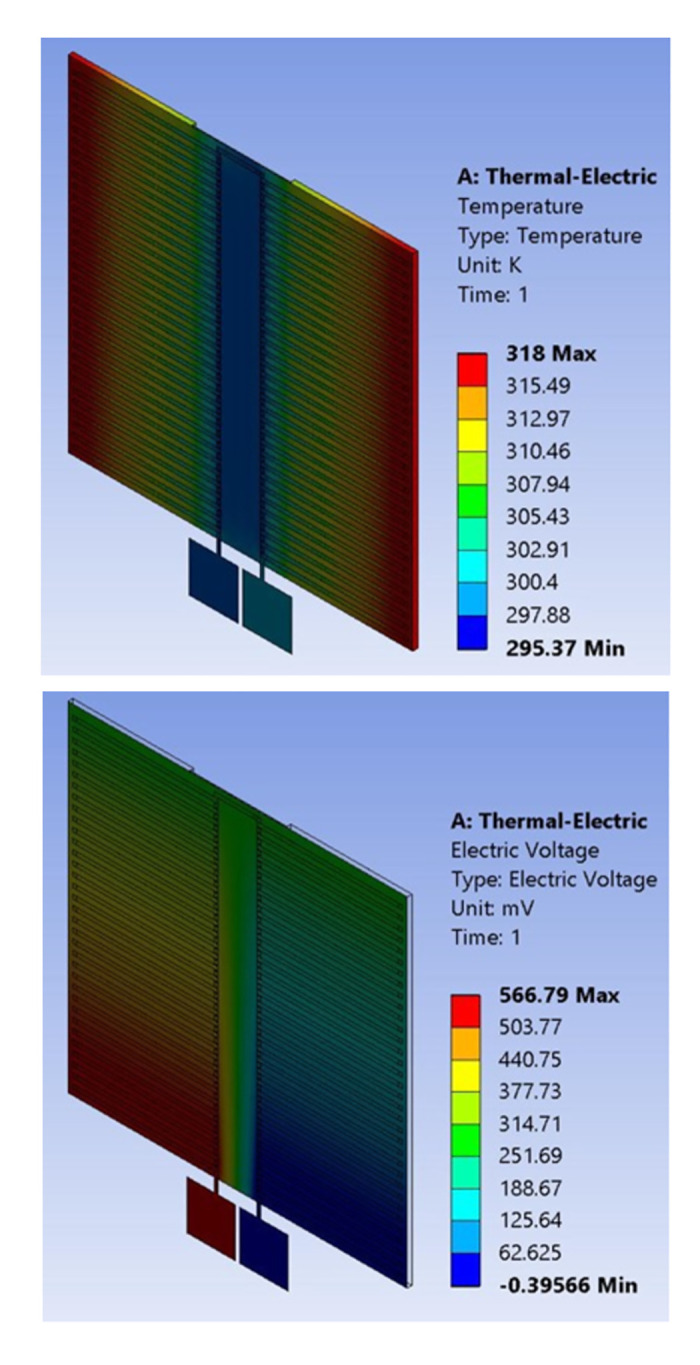
Temperature and Voltage distributions at ΔT = 20K: (a) Temperature, (b) Voltage.

This relation can be confirmed by the voltage distribution in Fig 3B. It can be seen that the voltage value is minimum (blue) at the negative terminal and progressively increases to maximum (red) at the positive terminal.

The temperature is high on the hot side and gradually reduces to a minimum at the cold side of the module as shown in Fig 3A.

### 3.2. Voltage against ΔT

**Fig 4:**
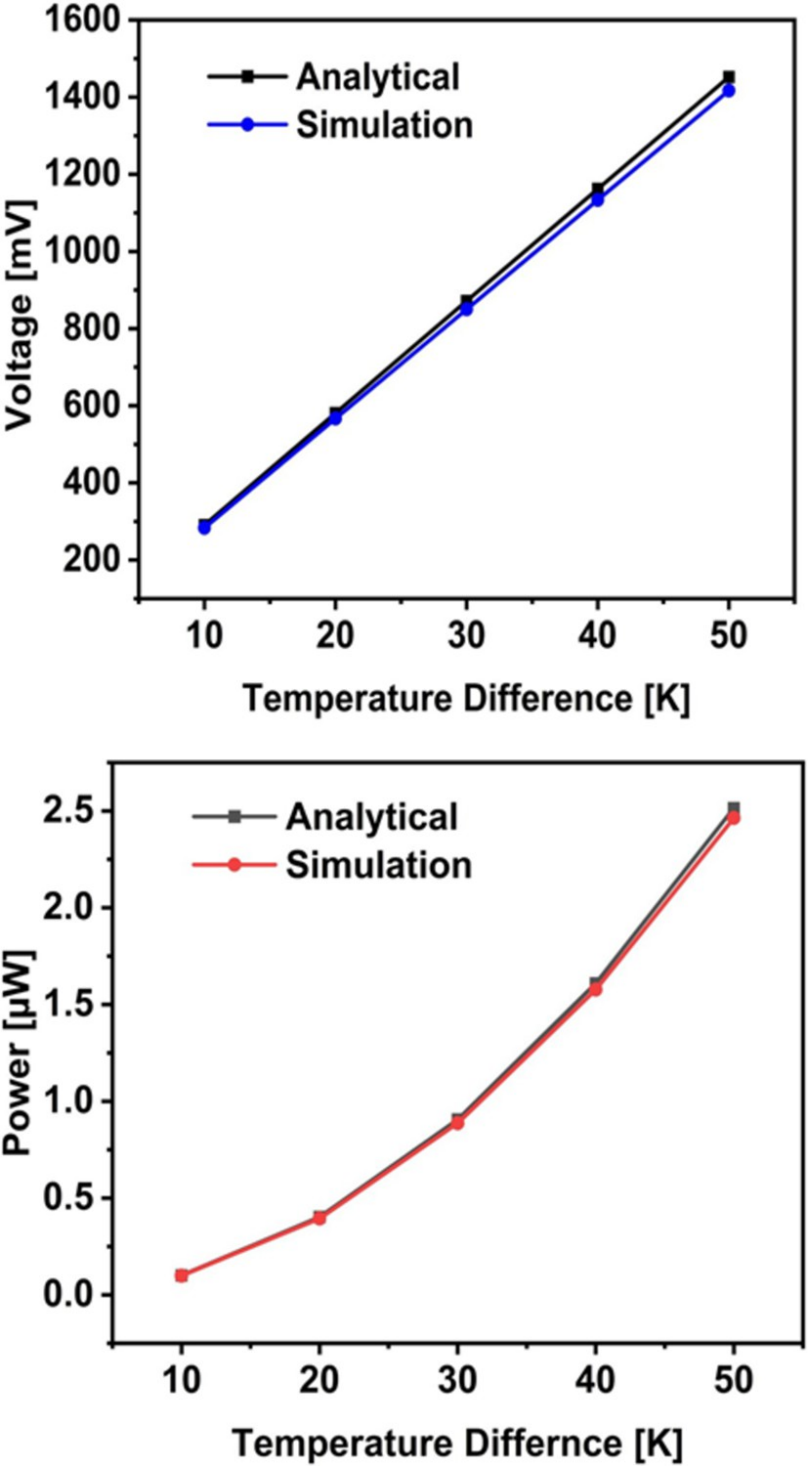
Analytical Vs Simulation results: (a) Voltage, (b) Power.

This was investigated by fixing the cold side temperature and increasing the hot side temperature to simulate different practical conditions. As shown in Fig 4A, the voltage increases as the temperature difference across the module increases. This is in agreement with Eq 1.

### 3.3. Power against ΔT

The maximum power is obtained when the internal resistance of the module is equal to the external load. The internal resistance of the generator obtained from the analytical study of this design was used to compute the power. As shown in Fig 4B, the power increases with increasing temperature difference, which is also in agreement with Eq 6.

### 3.4. Simulation versus analytical model

A mathematical model of the proposed design was developed and comprehensively analyzed in our previous work on micro thermoelectric generator design [13]. The power of the generator in terms of geometric terms is expressed as:

$$P_{TEG}=\frac{N{\left(\alpha \Delta T\right)}^{2}}{4(\frac{\rho_{a}l}{wt}+\frac{\rho_{b}l}{wt}+\frac{\rho_{a}l_{c}}{w_{c}t}+\frac{\rho_{b}l_{c}}{w_{c}t}+\frac{\rho_{b}l_{ch}}{wt_{ch}}+\frac{\rho_{b}l_{ch}}{w_{c}t_{ch}})}$$ (9)

The denominator in brackets is the internal resistance of the generator expressed in terms of the geometry where *ρ*_*a*_ and *ρ*_*b*_ are the electrical resistivities of n-type and p-type materials. l, w, and t are the length, width, and thickness of the thermoelectric films, while *l*_*c*_ and *w*_*c*_ are the length and width of the contacts. *l*_*ch*_ and *t*_*ch*_ are the length and thickness of the contact holes. The number of pairs, N, is given by:

$$N=\frac{0.07}{w+g}$$ (10)

Where *w* is the metal width and *g* is the gap between two pairs, expressed as g = 2 *l*_*c*_−*l*_*ch*_.

The thermal gradient across the TEG was varied and the corresponding output voltage and power were obtained.

### 3.5. Performance comparison with other designs

A single-layer planar TEG with design dimensions similar to those of the double-layer type was simulated under identical conditions. Also, a vertical topology TEG with dimensions of the commercial bulk type (TEC1-12706) was adopted and simulated under similar boundary conditions using the steady-state thermal-electric module in Ansys. The normalized values of the voltage and the power show the multi-layer structure suggested in the work is better than the single-layer type. The vertical type has a much higher power per unit area compared to the planar type. This is due to a high internal resistance exhibited by the planar type. The normalized values of voltage and power are summarized in Tables 1 and 2.

**Table 1:** Performance comparison of three different module configurations.

Module Type	Voltage (mV/cm2)	Power (W/cm2)
Double layer	69.4	7.2x10−8
Single layer	11.1	3.6x10−9
Vertical (commercial bulk-type)	85.2	3.6x10−3

**Table 2:** Comparison between paper and acrylic.

paper		acrylic	
pros	cons	pros	cons
self-sticking and easy to secure onto the substrate	not reusable	Pros reusable	cons
it is thin, less possibility of shadow deposition			requires tape to secure onto the substrate

## 4. Micro-fabrication process and experimental setup

The process starts with a clean silicon (Si) of 500 μm thickness with a layer of silicon dioxide (SiO_2_). Backside etching was achieved using the CO_2_ laser machine. The process is summarized as follows.

Step 1: Clean the Si wafer.

Step 2: Backside etching to create a diaphragm.

Step 3: Fit mask 1 and deposit the first metal (NiCr). Then remove the first mask to prepare for the second deposition.

Step 4: Apply mask 2 to deposit the second metal.

**Fig 5:**
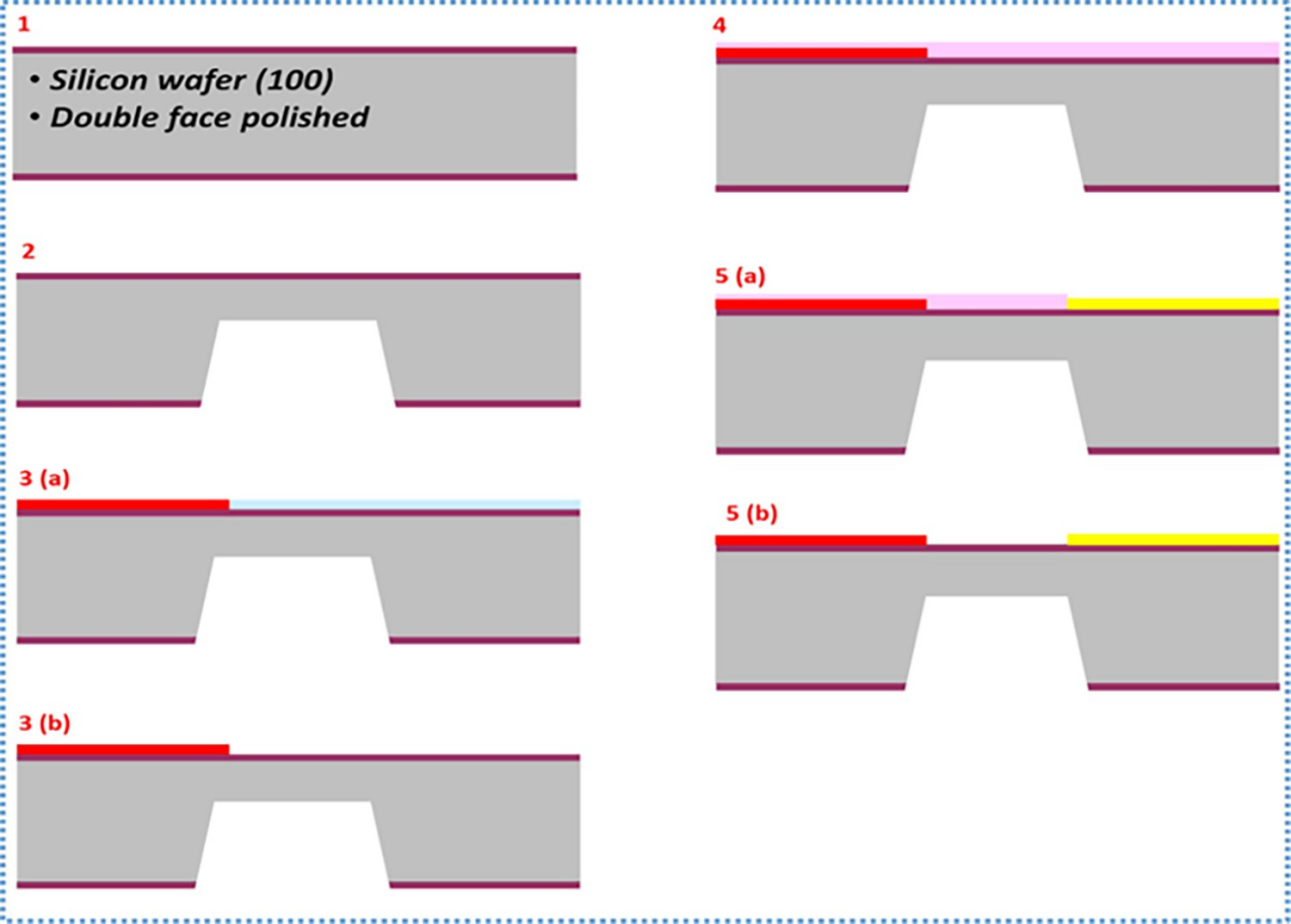
Fabrication procedure schematic of a single layer device.

Step 5: Deposit the second metal (CuNi). Remove mask 2 to have the final device in step 5 (a) in Fig 5.

### 4.1. Substrate preparation

**Fig 6:**
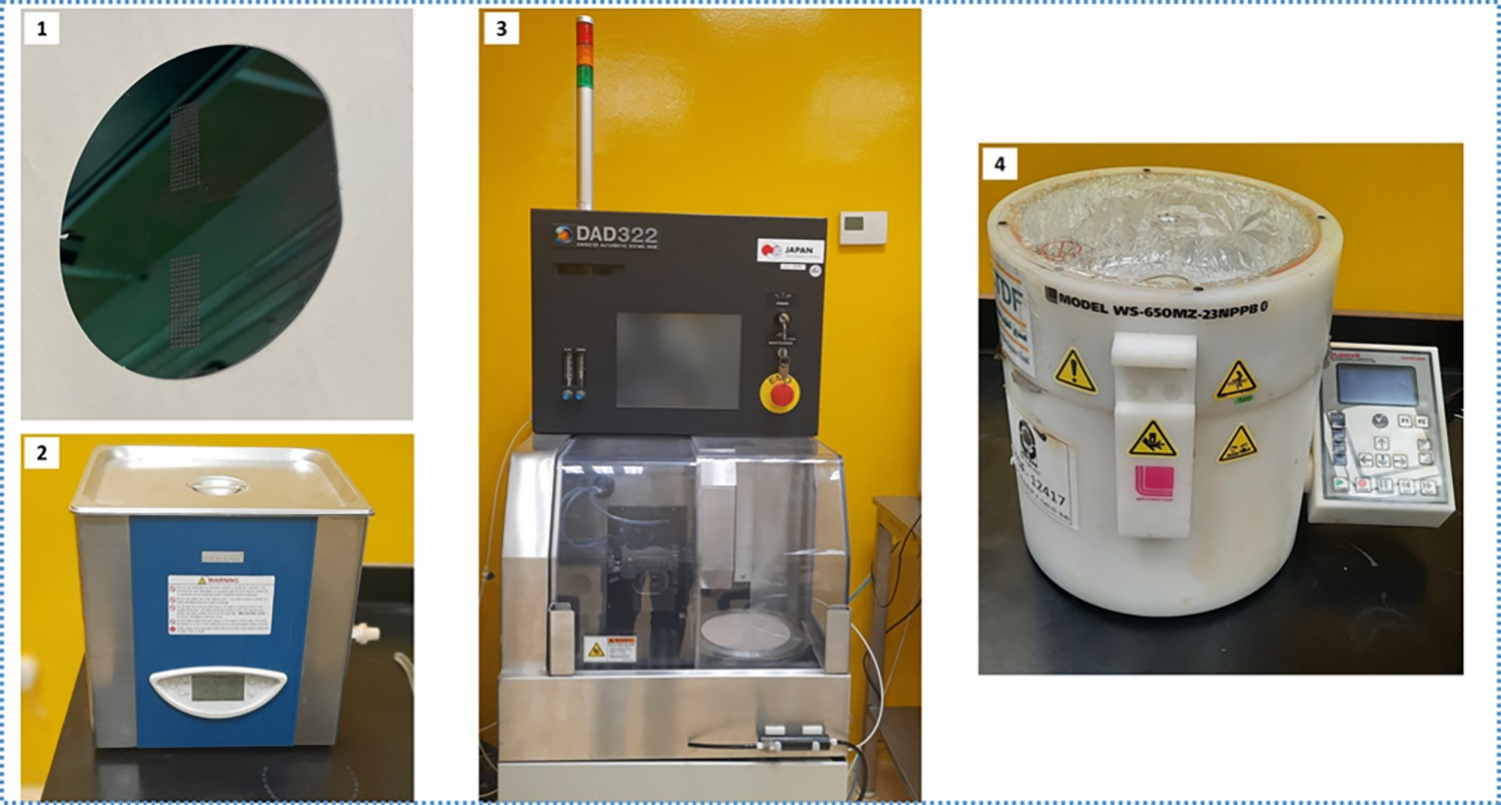
Substrate preparation: (1) Silicon wafer, (2) Ultrasonic cleaner (LABSONIC LBS2-4,5), (3) Dicing saw (DAD322) and (4) Spin coater (WS-650-23B).

The silicon wafer (100) of diameter 10 cm and thickness of 500 μm was used as the substrate. The effective substrate area used was 70x70 mm^2^, which is the maximum possible area of a quadrilateral enclosed in a circle of diameter 100 mm. First, the wafer was split into four equal parts using the dicing machine (DISCO-DAD-322) (Fig 6(3)), to allow for the fabrication of four devices of size 35x35 mm^2^. The wafer pieces were cleaned using acetone in a glass container and placed under the ultrasonic cleaner (LABSONIC LBS2-4,5) (Fig 6(2)) for 20 minutes. This was followed by methyl alcohol in a glass container also placed under the ultrasonic cleaner for 15 minutes. Lastly, the substrates were cleaned with deionized water in a glass container using the ultrasonic cleaner for 15 minutes. They were then dried using the spin coater (Laurell WS-650-23B) (shown in Fig 6(4)).

### 4.2. Shadow mask preparation

The process is developed and discussed for both paper and acrylic as follows.

#### 4.2.1. Paper mask

**Fig 7:**
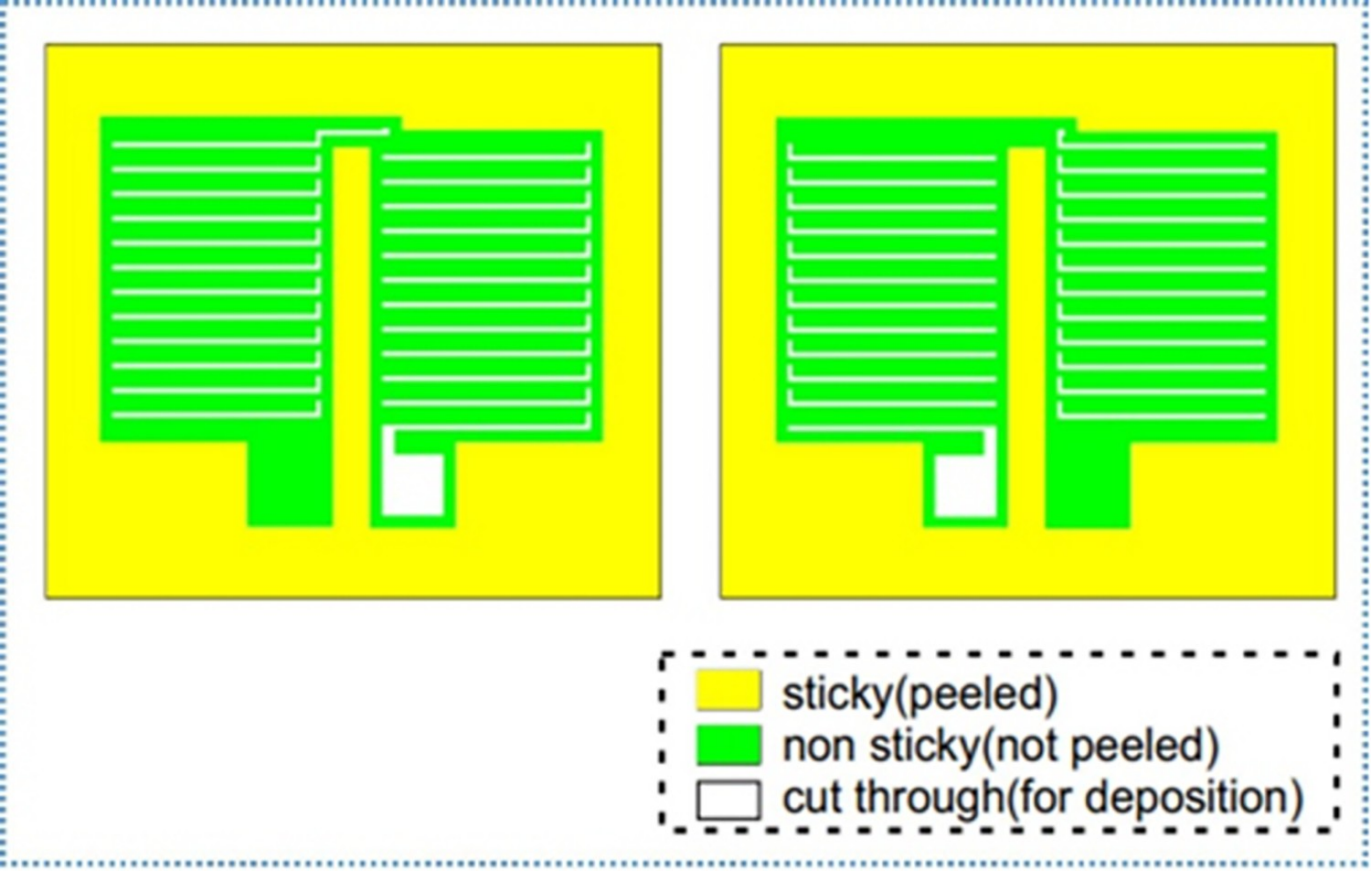
Illustrating the use of sticky paper from the backside.

Paper can be assumed abundant and easily available anywhere, anytime. Both normal (non-sticky) and sticky paper locally available in Egypt were used in this study. Sticky paper provided the advantage of self-sticking and better adhesion of the mask to the substrate. However, it had a challenge of residual glue which may affect the adhesiveness and connectivity of the subsequent depositions. Another challenge was peeling off part of the already deposited metal (first deposition) while removing the second mask after the second deposition. These shortcomings could easily be solved by the use of normal paper (non-sticky) and acrylic but also had the drawback of difficulty in securing the mask to the substrate. This was better solved by:

- Inverting the sticky paper and cutting the desired pattern from the back (non-sticky side).
- Cutting through both layers of the paper (sticky and non-sticky sides) for the desired pattern.
- Lastly, cutting and peeling off only the non-sticky layer at the periphery of the mask and other pre-determined areas to achieve self-sticking of the mask onto the substrate as illustrated in Fig 7.

#### 4.2.2. Procedure description

**Fig 8:**
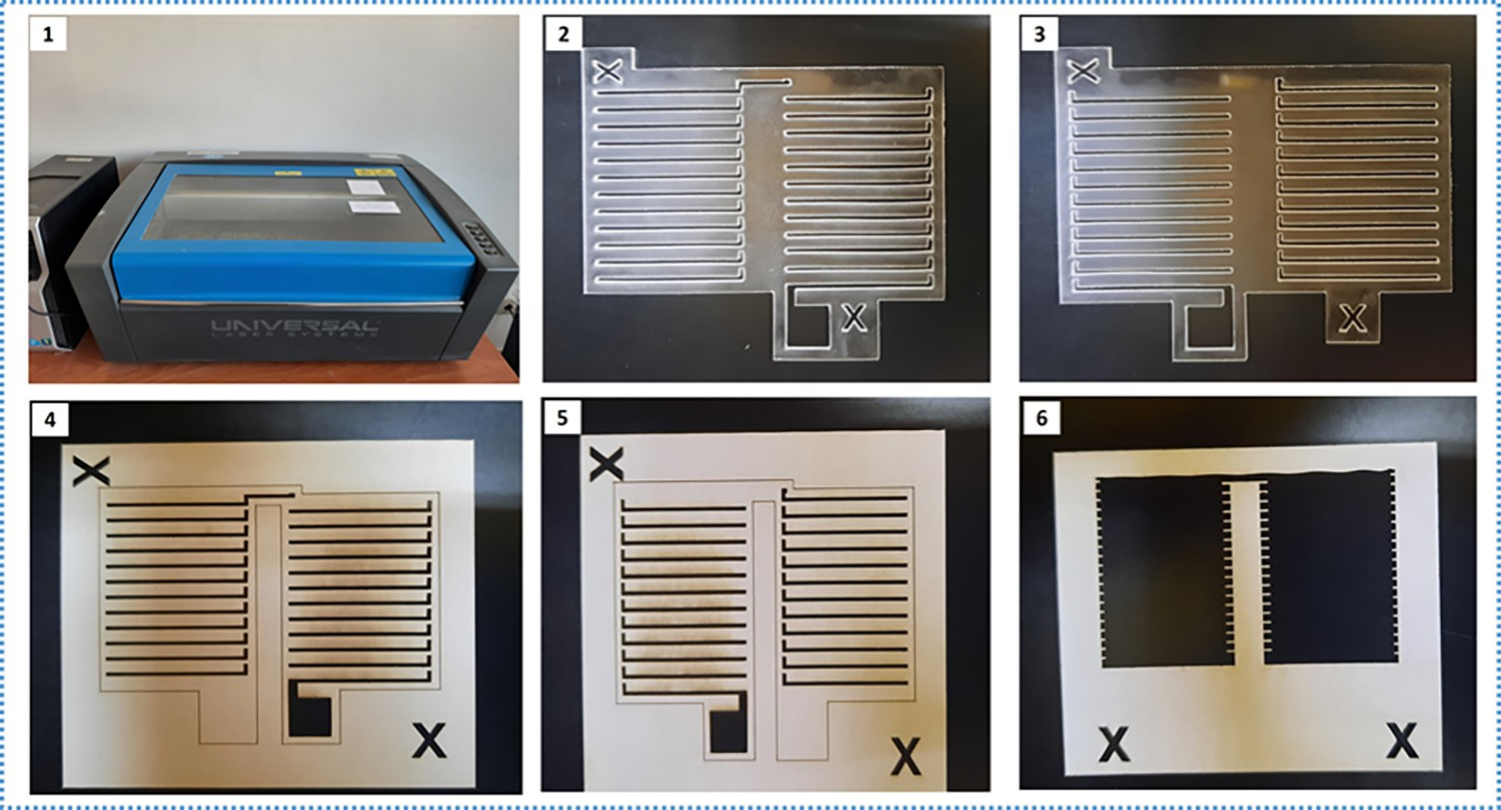
Shadow Mask preparation: (1) CO2 laser cutting machine (VLS 3.5), (2–3) Acrylic masks, and (4–6) Paper masks.

First, the desired micro-patterns were designed using the Corel DRAW software and then cut out on paper by direct laser writing using a Universal Laser System (VLS 3.5) shown in Fig 8(1). The machine has maximum power, speed, and resolution of 30 W, 250 mm/s, and 1000 pulses per inch (PPI), respectively. The machine has two methods of cutting i.e., the raster and vector modes. Both modes were studied to obtain a suitable one for this application. To ascertain the best conditions that would give the target mask dimensions, particularly the metal width, trial patterns were first cut. In each case, the cutting power and speed were varied, one at a time until the best combination(s) were obtained. The trial patterns were studied under a fluorescence microscope to determine the dimensions and quality of the edges.

*4*.*2*.*2*.*1*. *The raster mode*. Different combinations were tried, and observations were made as follows:

- High power and low speeds. The laser applies more heat at any one point for a longer time causing more burning at the edges of the patterns. This results in wider pattern dimensions (film width) and a much poor finish along the edges.
- Low power and high speeds. Applies less laser power for short times and could not sufficiently cut through the material (paper). With the speed fixed at 100%, the laser could only cut through both layers of the sticky paper starting at 60–100% of the power.
- High powers and high speeds. Proved to give the best combinations. High precision and edge quality were obtained at high cutting power with medium to high speeds. The laser power and speed set at 100% and 100% respectively of the maximum values gave the best possible quality and dimensions of the mask.
- Low power and low-medium speeds such as 30–40% were also not appropriate for raster mode.

It can be concluded that the raster mode is appropriate for use at high laser power and speeds.

4.2.2.2. The vector mode.

**Fig 9:**
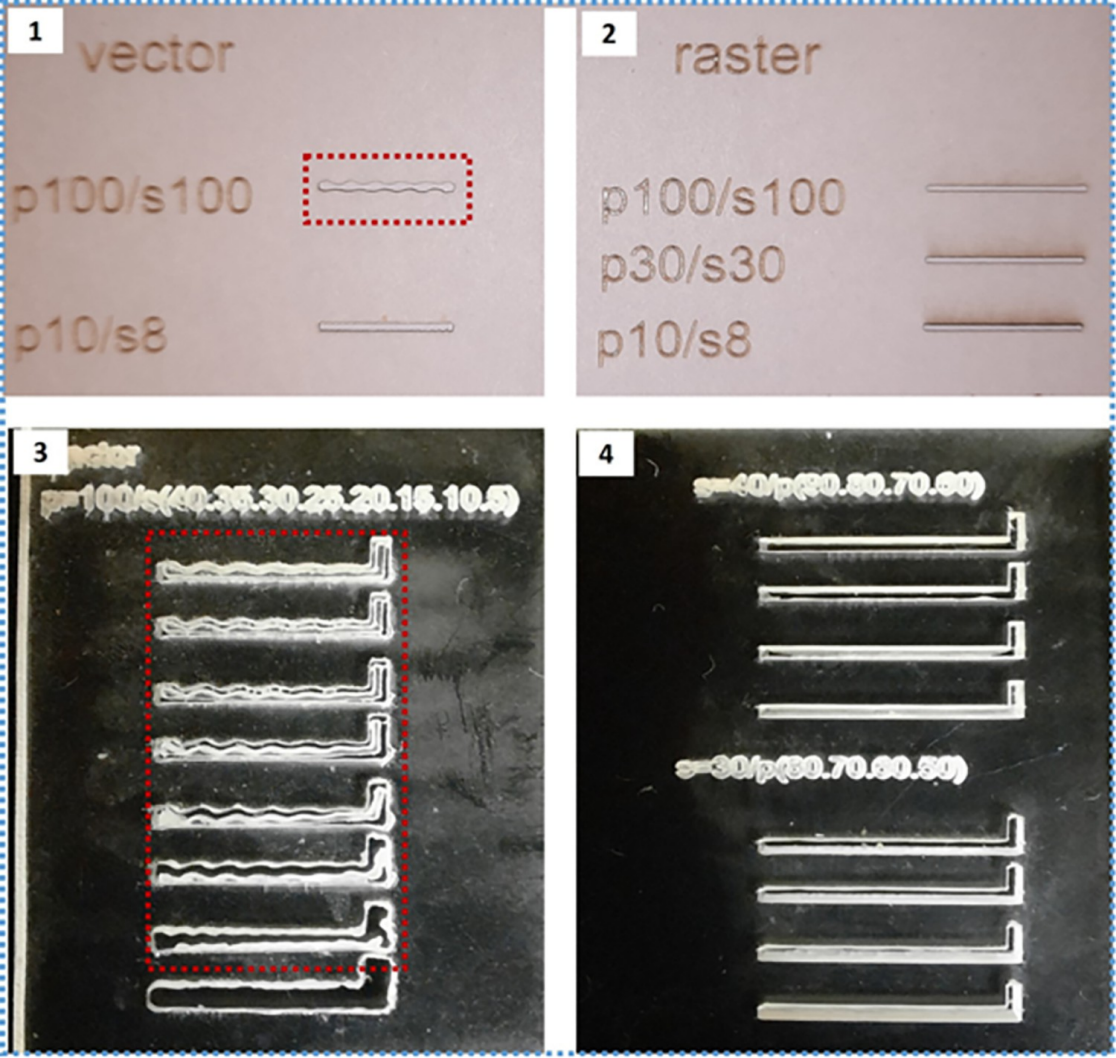
Shadow Mask optimization study using different laser cutting combinations: (1) Paper mask using vector mode, (2) Paper mask using raster mode, (3) Acrylic mask using vector mode, and (4) Acrylic mask using raster).

- High power and low speeds: The laser applies more laser heat for a longer time at the edges of the patterns causing wider pattern dimensions.
- Low/high power and high speeds. For both low and high power, the vector mode creates significant ripples, as can be seen in Fig 9(1) and 9(3), along the edges of the patterns while operating at high speeds.
- Low power and low speeds. These combinations proved ideal for vector mode. The power and speed at 10% and 8% respectively provided the best cutting combination in the vector mode. Generally, the vector mode is appropriate for use at low speeds since high speeds create ripples along the edges of the desired patterns.

The raster mode was selected at power and speed set at 100% and 100% respectively of the maximum values in the preparation of the final paper mask that was used in the fabrication of the device. This is because the raster had better results at its best conditions compared to the vector mode at its best conditions. However, the vector mode equally has good enough results and can be recommended for use in preparing the paper (sticky paper) masks.

#### 4.2.3. Acrylic mask

A 1 mm acrylic sheet was used. A small section of the sheet was placed into the working area of the Universal Laser System, VLS 3.5. Micro-patterns already designed in CorelDRAW software were cut out on the acrylic sheet. The same methodology applied in the use of paper was employed in the study of the use of acrylic as a shadow mask for the fabrication of micro thermoelectric generators.

*4*.*2*.*3*.*1*. *The raster mode*. Different combinations were studied by fixing the power and varying the speed, or vice versa and observations were made as follows:

- Fixed speed: We fixed the speed at 100% and varied the power from 100–80%, the sample could not cut through at all.
- Constant power. We fixed the power at 100% and varied the speed from 100% to 10%, the acrylic sample started to cut through at a speed of 50% and below, but at 50%, the width is narrower than the design width and it is wider at 20% and 10%. It was also observed that the quality of the finish along the pattern edges reduces as the speed reduces, being worst at 10%. High power (100%) and high speeds did not cut through at all, while high power (100%) and lower speeds produced wider widths and poor edge finishes. Hence, medium speeds and power slightly less than 100% could be ideal for the acrylic mask.
- Next, we fixed the speed at 30% and 40% and, in each case, varied the power from 50–90%, the combination of the speed at 40% and power at 90% gave the best results.

*4*.*2*.*3*.*2*. *The vector mode*. Vector mode, just as observed in the study of the paper mask, cannot be used at high speeds as it creates ripples along the periphery of the patterns.

- First, we fixed the power at 15% and varied the speed from 8–1%. Even at lower speeds, the sample still exhibited ripples for speeds 8, 7, and 6% and only disappeared from the speed of 5% and below. Moreover, speeds 8, 7, and 6% could not cut through the sample.
- Next, we fixed the speed at 5% and varied the power from 20% to 5%. The best combination was found to be 5% and 10% for the speed and power respectively.

**Fig 10:**
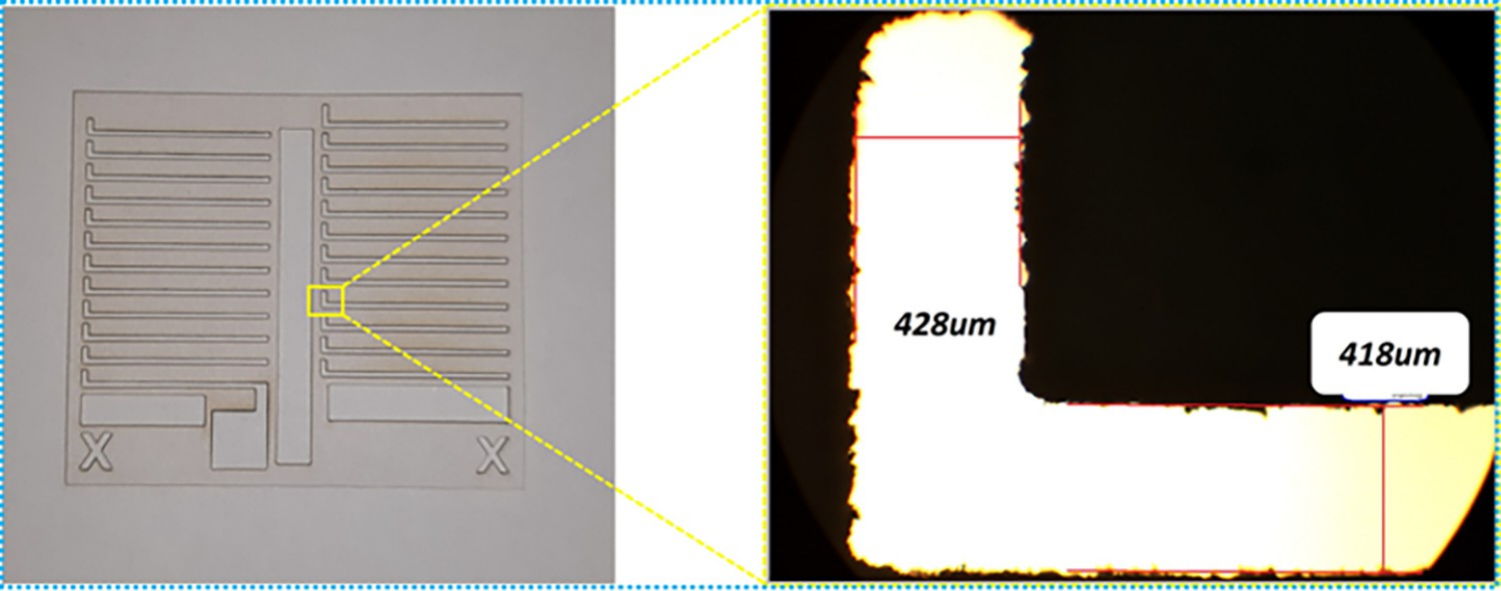
Prepared shadow mask (paper).

The vector mode was selected for the preparation of the final acrylic mask as its performance in terms of precision and edge quality was slightly better than the raster mode. Also, important to note that the vector mode is faster than the raster for all conditions. It is also important to note that the material thickness affects the cutting speed as can be seen in both cases (vector and raster) for acrylic and paper. The speed reduces as the material thickness increases. The acrylic sheet used was 1mm thick while the paper was 100–150 μm. For example, for the best vector conditions for paper and acrylic, for the same power, the speed for acrylic was less than that for the paper mask. In raster mode, even if the power was slightly reduced for acrylic, the speed was much less (40%) compared to 100% for the paper mask. The final masks were checked under the microscope to verify the dimensions of the metal legs and contacts, as can be seen in Fig 10.

### 4.3. Sputtering deposition

**Fig 11:**
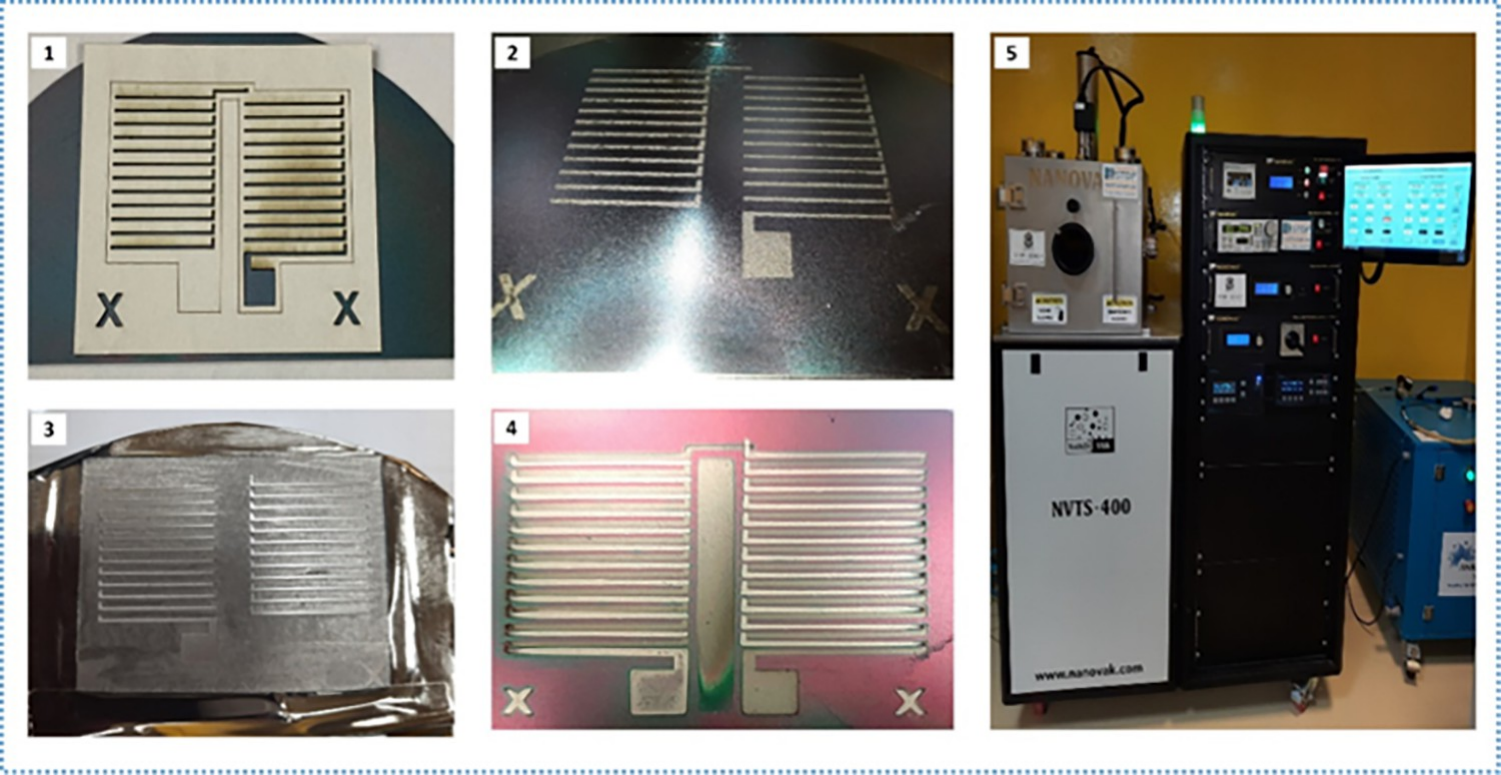
Deposition and fabrication of the device (single-layer): (1) First mask fitted onto the substrate, (2) Metal 1 deposited circuit, (3) Second mask fitted, and (4) Second metal deposited and final device.

Chromel (NiCr) and Constantan (CuNi) targets were deposited to form the p- and n-type TE legs respectively using a physical vapor deposition technique by RF magnetron sputtering using the NVTS-400-2TH2SP deposition machine shown in Fig 11(5). High purity 99.99% NiCr and CuNi targets with a diameter of 2.0 inches were used as the source materials for p-type and n-type films respectively. The target is fixed at a tilted angle relative to the substrate. The substrate is rotated at a rate of 5 rpm to prevent shadow effects and ensure uniform film deposition. A vacuum of approximately 1x10^−6^ Torr was achieved in the deposition chamber before starting the deposition in each case. A proper vacuum pressure, preferably in the order of micro-Torr ensures that any gas residues and impurities in the deposition chamber are removed and guarantees a higher deposition rate. Argon (Ar) sputtering gas is subsequently introduced at a rate of 5 sccm (standard cubic cm/min). The highest deposition rate obtained was 1 A/s (angstrom per second).

The power was set at 300 W and uniform films of NiCr and NiCu were obtained to fabricate the micro energy harvester.

## 5. Experimental results

**Fig 12:**
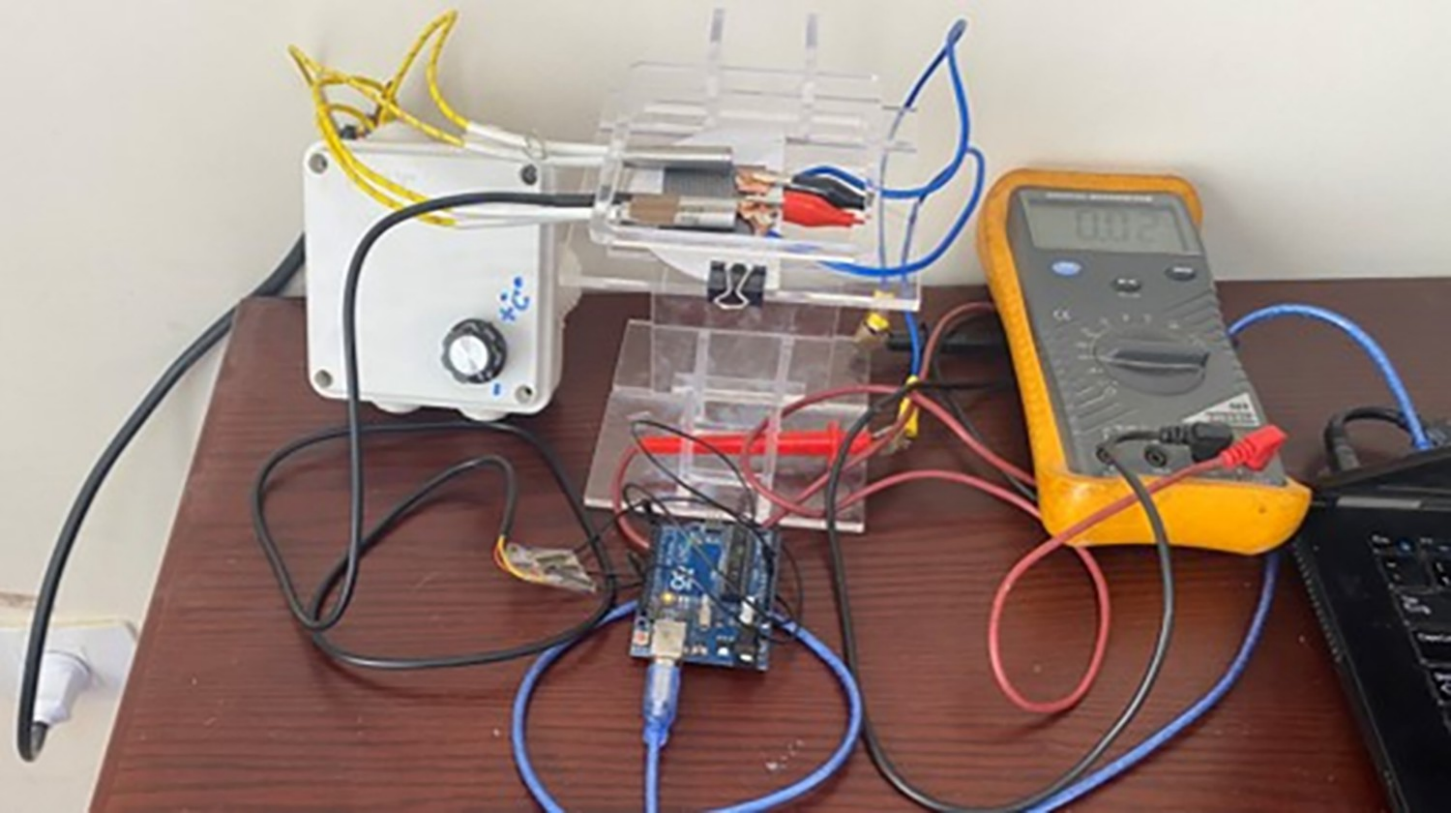
Experimental setup.

Fig 12 shows the testing setup with the micro thermoelectric generator on top of a stand with all the electric connections for measuring resistance, voltage, and temperature. The hot side temperature was achieved with the use of two heaters controlled by a solid-state relay and a variable resistor. The cold side temperature was obtained by using an ice block as the hot side temperature was increased for higher thermal gradients.

**Fig 13:**
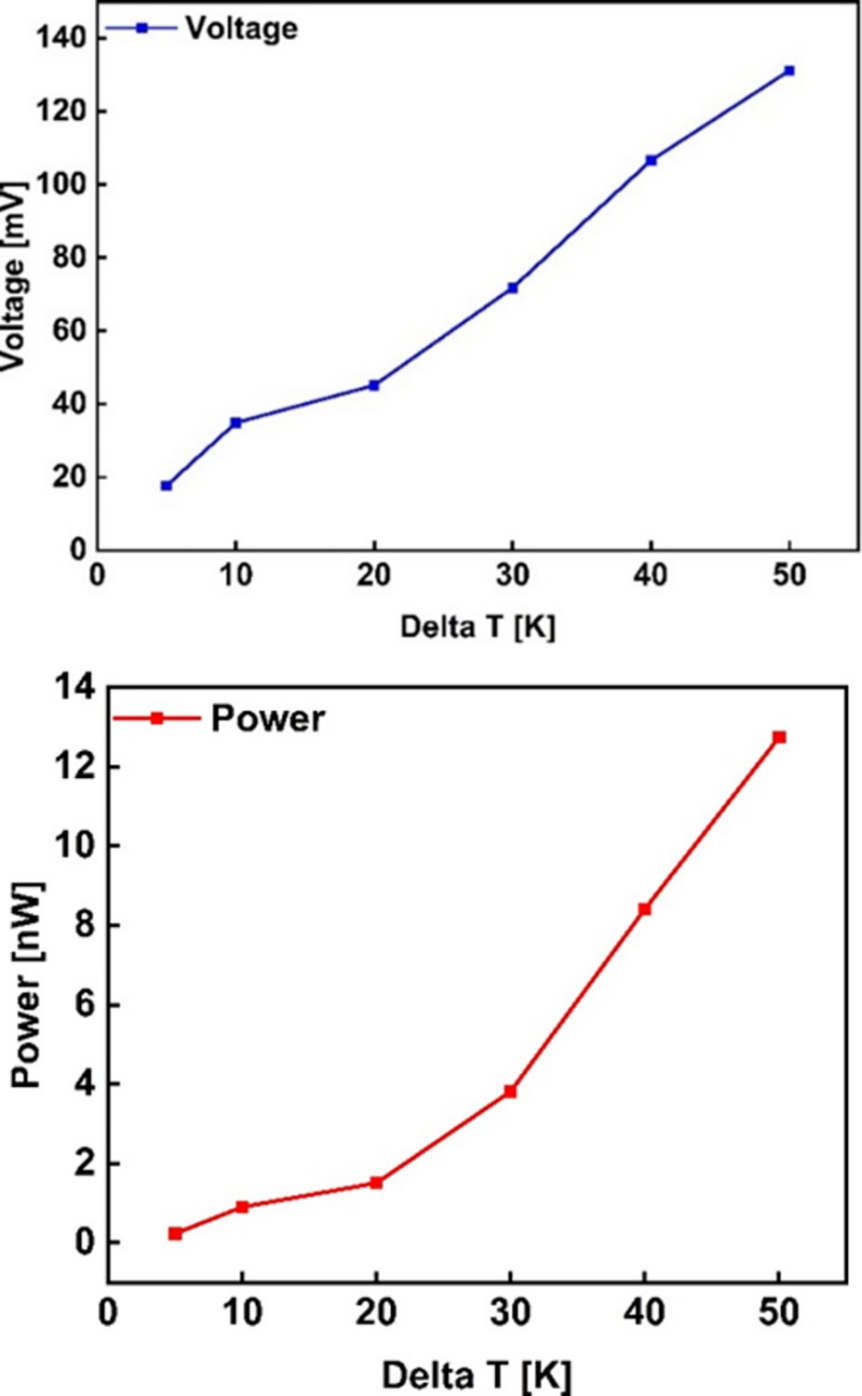
Temperature difference versus (a) Voltage and (b) Power.

**Fig 14:**
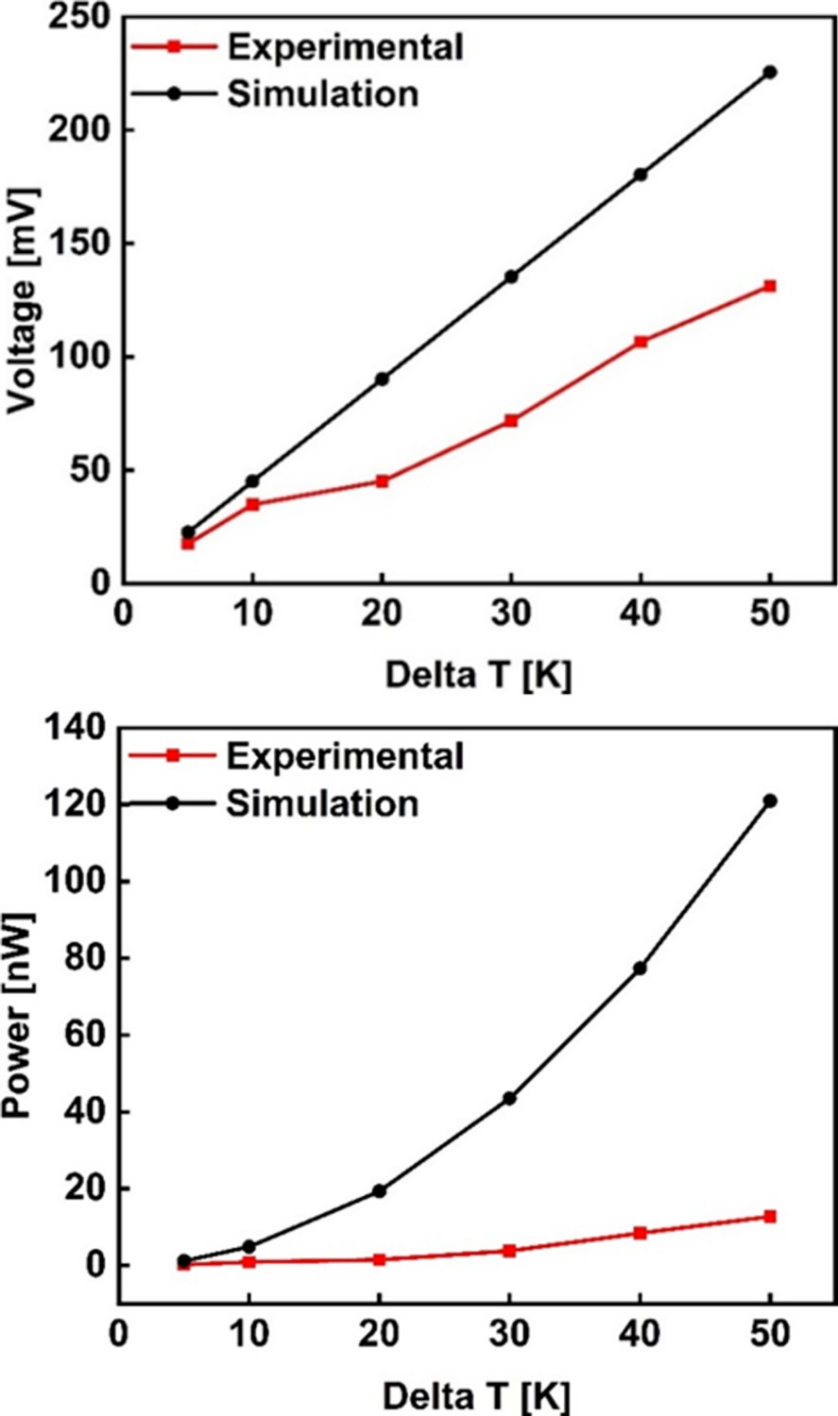
Experimental vs Simulation: (a) Voltage, (b) Power.

The open circuit voltage was measured by varying the hot-side temperature to get different temperature gradients, and the corresponding values of power were obtained as shown in Fig 13. The voltage has a linear relationship with the temperature difference. As shown in Fig 13A, it increases as the temperature gradient is increased and this is in agreement with Eq 1 for a thermocouple and Eq 7 for a TEG of N pairs. The power output is directly proportional to the square of the temperature difference. Hence, it increases as the temperature difference is increased as depicted in Fig 13B. This is also in agreement with Eqs 6 and 8 for a thermocouple and a TEG of N pairs respectively. When compared with the simulation results, the experimental results follow a similar trend as seen in Fig 14. However, a big percentage error in the power output was noted between the two sets of results. This could be attributed to a number of reasons, but not limited to: the interfacial problems, such as the electrical contact resistance, thermal contact resistance at the metal-semiconductor interface, as well as element diffusion; variations in material properties arising from manufacturing inconsistencies and temperature dependence of materials; and micro-fabrication complexities such as non-uniformities in the film dimensions, and grain boundary defects. Additionally, since experimental setups are conducted in the real-world environments, boundary conditions such as temperature gradients and heat loss, among others, considerably differ from simulation conditions. For instance, the internal resistance of the fabricated device was more than 3 times the module resistance obtained from the simulations. These disparities can be mitigated by post-deposition treatment such as annealing. Post-heat treatment increases the electrical transport and thermoelectric properties of the films thereby improving the power factor of the as-deposited device. Furthermore, material characterization including extracting material properties from the as-built TEG for accurate input data into the simulations could help reduce the disparity gap.

## 6. Conclusion

This paper has successfully achieved the design and fabrication of a planar-based micro-thermoelectric generator for energy harvesting applications using thin-film technology. The device was analyzed using the steady-state thermoelectric module in Ansys. The simulation study achieved an open-circuit voltage of 1417 mV and a power output of 2.4 μW for a temperature difference of 50 K. When compared with the results of the analytical model, there was an error of 2.44% and 2.03% in the open circuit voltage and power respectively. The study has introduced the use of paper and acrylic to prepare shadow masks, instead of photoresists and photo-lithography processes. The proposed approach simplifies the fabrication process of planar TEGs. For instance, with the masks ready, the process of fabricating the proposed planar double-layer TEG was reduced to three major steps, while that of a single-layer planar type was only two major steps. Experimental results show that the fabricated generator is capable of generating power from a small range of temperature gradients. The fabricated single-layer device can produce 131 mV when subjected to a 50 K temperature difference. This could produce about 0.12 μW, which is enough to power some wearable sensors/devices.
